# Developing Prediction Equations and a Mobile Phone Application to Identify Infants at Risk of Obesity

**DOI:** 10.1371/journal.pone.0071183

**Published:** 2013-08-07

**Authors:** Gillian Santorelli, Emily S. Petherick, John Wright, Brad Wilson, Haider Samiei, Noël Cameron, William Johnson

**Affiliations:** 1 Bradford Institute for Health Research, Bradford Royal Infirmary, Bradford, United Kingdom; 2 iED, Bradford, United Kingdom; 3 School of Sport, Exercise and Health Sciences, Loughborough University, Loughborough, United Kingdom; 4 Unit for Lifelong Health and Ageing, Medical Research Council, London, United Kingdom; University Hospitals of Geneva, Switzerland

## Abstract

**Background:**

Advancements in knowledge of obesity aetiology and mobile phone technology have created the opportunity to develop an electronic tool to predict an infant’s risk of childhood obesity. The study aims were to develop and validate equations for the prediction of childhood obesity and integrate them into a mobile phone application (App).

**Methods and Findings:**

Anthropometry and childhood obesity risk data were obtained for 1868 UK-born White or South Asian infants in the Born in Bradford cohort. Logistic regression was used to develop prediction equations (at 6±1.5, 9±1.5 and 12±1.5 months) for risk of childhood obesity (BMI at 2 years >91^st^ centile and weight gain from 0–2 years >1 centile band) incorporating sex, birth weight, and weight gain as predictors. The discrimination accuracy of the equations was assessed by the area under the curve (AUC); internal validity by comparing area under the curve to those obtained in bootstrapped samples; and external validity by applying the equations to an external sample. An App was built to incorporate six final equations (two at each age, one of which included maternal BMI). The equations had good discrimination (AUCs 86–91%), with the addition of maternal BMI marginally improving prediction. The AUCs in the bootstrapped and external validation samples were similar to those obtained in the development sample. The App is user-friendly, requires a minimum amount of information, and provides a risk assessment of low, medium, or high accompanied by advice and website links to government recommendations.

**Conclusions:**

Prediction equations for risk of childhood obesity have been developed and incorporated into a novel App, thereby providing proof of concept that childhood obesity prediction research can be integrated with advancements in technology.

## Introduction

Childhood obesity is one of the most daunting global public health threats [1], with the projected cost to the UK National Health Service (NHS) estimated to be as high as £9.7 billion per year by 2050 [2]. The secular trend of increasing prevalence and earlier onset of childhood obesity [3]–[5] will have long term implications for health care because obesity tracks through the life course [6], [7], increasing risk for a plethora of adverse health outcomes [8]. A key to improving the future health of the nation must lie in the prevention of childhood obesity as well as the treatment of its downstream sequalae.

The aetiology of childhood obesity is complex [9], but two simple measures, greater birth weight and accelerated weight growth during infancy, have consistently been shown to increase risk of childhood obesity [10]–[14]. In a recent meta-analysis, Druet et al [15] found that the risk of childhood obesity increased two-fold with each one unit increase in weight z-score between birth and one year (odds ratio (OR) 1.97, 95% confidence interval (95% CI) 1.83–2.12), with the risk of adult obesity increasing by 23% (OR 1.23, 95% CI 1.16–1.30). Further, the combination of birth weight, infant weight gain, and maternal body mass index (BMI) had a good ability to predict the risk of an infant becoming obese in childhood, with an area under the curve (AUC) of 77% (95% CI 74–80%).

The advancing knowledge of risk factors for childhood obesity and in mobile phone technology has created the opportunity to develop an electronic tool that predicts during infancy an individual’s risk of becoming obese. In the UK, growth monitoring in infancy is part of routine National Health Service (NHS) care [16], thereby making the integration of an obesity risk tool with routine practice an achievable goal. An example of a paper-based tool for predicting obesity has been published [17], but it lacks the sophistication and the usability necessary for practice. For example, it only includes one prediction equation, thereby limiting its use to one age in infancy, and it requires the user to do the calculations. More recently, a web-based risk calculator for predicting childhood obesity in newborns has been developed [18], which performs the background calculations and estimates the risk of obesity as a percentage. Whilst this is a great improvement on the paper-based tool, extensive input data is required for variables which may not be available at assessment, and this may unfortunately limit its usability. It is the advent of smartphones and mobile devices such as tablets that really have the potential to revolutionise this type of clinical prediction tool. The uptake of smartphones is remarkable: in 2011 81% of US physicians used a smartphone [19], thus a mobile phone application (App) that can predict childhood obesity has the advantage of being instantly available to thousands of users.

The present study aims to (1) develop prediction equations which can be used during infancy for the early identification of risk of childhood obesity, (2) validate the prediction equations internally using statistical methods and externally in a different population, and (3) integrate the equations into a novel user-friendly App.

## Methods

### Sample

The sample comprised 1868 participants in the Born in Bradford (BiB) birth cohort study (http://www.borninbradford.nhs.uk/) [20], [21], of whom 804 were White British (422 boys, 382 girls) and 1064 were South Asian (540 boys, 524 girls). Briefly, BiB is a longitudinal multi-ethnic birth cohort study which recruited 12,453 women comprising 13,776 pregnancies recruited at approximately 28 weeks gestation between 2007 and 2010. The study aims to examine the impact of environmental, psychological and genetic factors on maternal and child health. Bradford is a city in the north of England with high levels of socio-economic deprivation and ethnic diversity. Similar to other cohorts, BiB has a subsample (BiB 1000, N = 1,735) whose data have been augmented by more detailed assessments than those conducted in the full cohort [22]. The parents of all participants gave informed written consent for the data collection, and ethical approval was granted by Bradford Research Ethics Committee (Ref 07/H1302/112).

### Data

Weight and length at six, 12, and 24 months of age were measured by trained study workers as part of the BiB 1000 assessment schedule. Weight in kilograms (kg) was assessed using Seca baby scales and length in centimetres (cm) using a standard neonatometer (both from Harlow Health Care, London UK). These data were supplemented by infant weight and length measurements collected by health visitors as part of routine NHS care. At the beginning of BiB, a measurement protocol/standard was produced and all health workers received training in anthropometry [23]. Test-retest reliability was subsequently assessed and technical errors of measurement were reported to be similar to those obtained by anthropometrists in research studies [23]. In addition, agreement between routine measurements and research measurements in a separate UK birth cohort study (ALSPAC) has been shown to be high [24], thereby providing justification for combining routine and research data in the present paper. A total of 3281 weight and length measurements were used in our analysis, 878 (26.8%) of which were research data and 2403 (73.2%) of which were routine data.

Data on childhood obesity risk factors were obtained from a number of sources. Maternal height (cm), ethnicity (*White British/South Asian (Pakistani, Indian, Bangladeshi and other South Asian*)), education (*<5 GCSE equivalent, ≥5 GCSE equivalent, ‘A’ level equivalent, Degree level equivalent, and other*), and smoking during pregnancy (*yes/no*)) were obtained from an administered baseline questionnaire completed at recruitment at approximately 28 weeks of gestation. Maternal weight (kg) at pregnancy booking (approximately 12 weeks gestation), gestational diabetes (yes/no), gestational age at birth (*<37 weeks/≥37 weeks*), gender (*male/female*), and birth weight (kg) were extracted from NHS maternity records. Maternal BMI (kg/m^2^) was calculated using weight measured at pregnancy booking and height from the baseline questionnaire.

### Development of Prediction Equations

Logistic regression was used to develop the prediction equations. Anthropometric data were converted to age- and sex-adjusted z-scores by comparison to the UK90 reference [25]. The outcome was “risk for childhood obesity” defined as a BMI greater than the 91st centile at age two years (± two months) and a conditional [26] weight z-score gain between birth and 2 years of age (± two months) greater than one centile band. Conditional weight gain was used because it accounts for starting size and regression to the mean. Predictor variables included sex, birth weight z-scores, conditional infant weight z-score gain from birth to assessment age (i.e. the age at which the infant would be assessed for risk), maternal BMI, and the other variables listed in the data section.

To ensure that the App could be utilised in children over a wide range of ages in infancy, we developed three series of equations, the first to be used at 6±1.5 months (equation 1), the second at 9±1.5 months (equation 2), and the third at 12±1.5 months (equation 3). The App would therefore be able to assess risk for childhood obesity in infants aged 4.5 to 13.5 months. Sample selection was based on complete covariate data, birth weight and weight/length data at age two years (±2 months) in addition to weight data in at least one of the three age periods when assessment would take place. The number of infants in each prediction equation differed slightly, but was always greater than 700 (Table 1).

**Table 1:** Characteristics of the development equation samples in the BiB cohort.

	Equation 1 sample	Equation 2 sample	Equation 3 sample
	N = 1022	N = 1528	N = 731
Source of data			
BiB 1000 data	310 (30.3)	281 (18.4)	287 (39.3)
Routine NHS data	712 (69.7)	1247 (81.6)	444 (60.7)
Baby’s sex			
Boys	538 (52.6)	785 (51.4)	368 (50.3)
Girls	484 (47.4)	743 (48.6)	363 (49.7)
Birthweight z-score, mean (SD)			
Boys	−0.57 (1.2)	−0.56 (1.2)	−0.58 (1.2)
Girls	−0.56 (1.2)	−0.52 (1.2)	−0.58 (1.2)
Weight change z-score, mean (SD)^a^			
Boys	0.07 (0.9)	0.06 (1.0)	0.02 (0.9)
Girls	−0.08 (1.0)	−0.06 (1.1)	−0.02 (1.1)
Infant BMI z-score at 2 years, mean (SD)			
Boys	−0.03 (1.1)	−0.03 (1.1)	−0.08 (1.1)
Girls	−0.08 (1.2)	−0.03 (1.2)	−0.01 (1.1)
Ethnicity			
White British	492 (48.1)	654 (42.8)	331 (45.3)
South Asian	530 (51.9)	874 (57.2)	400 (54.7)
Maternal BMI, mean (SD)	25.9 (5.6)	26.0 (5.6)	25.8 (5.5)
Mother’s education^b^			
<5 GCSE equivalent	190 (18.6)	330 (21.6)	142 (19.4)
≥5 GCSE equivalent	310 (30.3)	472 (30.9)	222 (30.4)
‘A’ level equivalent	172 (16.8)	233 (15.3)	124 (17.0)
Degree level equivalent	291 (28.5)	406 (26.6)	195 (26.7)
Other	59 (5.8)	87 (5.7)	48 (6.6)
Smoked during pregnancy^c^	136 (13.3)	201 (13.2)	97 (13.3)
Gestational diabetes^c^	76 (7.4)	113 (7.4)	58 (7.9)
Gestational age (<37 weeks)^c^	47 (4.6)	75 (4.9)	33 (4.5)
Obesity and rapid weight gain at 2 years^d^	83 (8.1)	121 (7.9)	61 (8.3)

a Weight change z-score from birth to 6, 9 or 12 months in equation 1, 2 and 3 samples respectively.

b GSCE = General Certificate of Secondary Education; A-level = Advanced level.

c Dichotomised *Yes/No* variable. Numbers are for *Yes.*

d Infant BMI >91^st^ centile and growth from birth to 2 years of age ≥1 centile band.

Values are numbers (percentages) unless stated otherwise.

All potential predictors were entered into backward stepwise multivariable models that retained predictors with a p-value <0.05. These models tested possible interactions of sex by birth weight, sex by conditional infant weight z-score gain, ethnicity by birth weight, and ethnicity by conditional infant weight z-score gain. Individual risk prediction scores were calculated using the coefficients (where α is the constant and β_1_ to β_k_ is a vector of predictors) from each of the three final prediction equations:



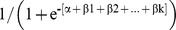
.

Sensitivity, specificity, and positive and negative predictive values (PPV and NPV, respectively) were calculated at risk score distribution cut-off points of 10%, 20% and 30% and area under the curves (AUCs) for the final logistic regression models were obtained to quantify the overall discrimination of the equations.

All analyses were conducted using Stata/SE v12 [27].

### Validation of Prediction Equations

Internal validity was assessed using bootstrapping methods [28]. One thousand repetitions were used with replacement from the original sample for each equation, and the final bootstrap models then applied to the original samples.

External validity was assessed by applying the equations to an external sample and calculating the AUCs. We used data from the Children in Focus (CiF) subsample of the Avon Longitudinal Study of Parents and Children (ALSPAC) [29]. ALSPAC recruited 14,541 pregnant women, and 1432 families attended at least one CiF clinic. Ethical approval for the study was obtained from the ALSPAC Ethics and Law Committee and the Local Research Ethics Committees. The sample sizes of the ALSPAC cohort obtained for the present study were: equation 1 (n = 7), equation 2 (n = 880), and equation 3 (n = 867). Due to insufficient numbers equation 1 could not be validated with the ALSPAC data.

## Results

Tables 1 and 2 describe the characteristics of the development (BiB 1000) and external validation (ALSPAC) samples respectively. The main difference between the samples from the two cohorts were that almost all the infants in the ALSPAC samples were of White origin (98%) compared to around 45% of infants of White British origin in the BiB 1000 cohort.

**Table 2:** Characteristics of the external validation samples in the ALSPAC cohort.

	Equation 2 sample	Equation 3 sample
	N = 880	N = 867
Baby’s sex		
Boys	481 (54.7)	470 (54.2)
Girls	399 (45.3)	397 (45.8)
Birthweight z-score, mean (SD)		
Boys	−0.10 (1.08)	−0.09 (1.06)
Girls	−0.01 (1.01)	−0.02 (1.01)
Weight change z-score, mean (SD)^a^		
Boys	0.01 (0.98)	0.30 (0.96)
Girls	−0.01 (0.94)	−0.35 (0.92)
Infant BMI z-score at 2 years		
Boys	0.18 (0.98)	0.17 (0.98)
Girls	0.25 (0.93)	0.26 (0.93)
Ethnicity		
White	865 (98.3)	852 (98.3)
Other	15 (1.7)	15 (1.7)
Maternal BMI, mean (SD)	23.4 (4.0)	23.4 (4.1)
Mother’s education^b^		
<5 GCSE equivalent	92 (10.5)	91 (10.5)
≥ GCSE equivalent	323 (36.7)	318 (36.7)
‘A’ level equivalent	240 (27.3)	236 (27.2)
Degree level equivalent	132 (15.0)	130 (15.0)
Other	93 (10.6)	92 (10.6)
Smoked during pregnancy^c^	150 (17.1)	146 (16.8)
Gestational diabetes^c^	3 (0.3)	2 (0.2)
Gestational age (<37 weeks)^c^	36 (4.1)	35 (4.0)
Obesity and rapid weight gain at 2 years^d^	84 (9.6)	84 (9.7)

a Weight change z-score from birth to 9 or 12 months in equation 2 and 3 samples respectively.

b GSCE = General Certificate of Secondary Education; A-level = Advanced level.

c Dichotomised *Yes/No* variable. Numbers are for *Yes.*

d Infant BMI >91^st^ centile and growth from birth to 2 years of age ≥1 centile band.

Values are numbers (percentages) unless stated otherwise. Equation 1 could not be validated due to insufficient numbers.

The prevalence of childhood obesity in the BiB 1000 and ALSPAC samples respectively was 8.1% in equation 1 (insufficient data in the ALSPAC sample), 7.9% and 9.6% in equation 2, and 8.3% and 9.7% in equation 3.

### Childhood Obesity Risk Prediction

#### Development model

Table 3 shows the factors that were significantly associated with the risk of childhood obesity at 2 years in the development models. The equation 1 sample revealed significant associations between risk of childhood obesity at 2 years and birthweight z-score, weight change z-score, maternal BMI, South Asian ethnicity and gestational age <37 weeks. The equation 2 sample saw significant associations with birthweight z-score, weight change z-score and maternal BMI. In the equation 3 sample, only birthweight z-score and weight change z-score were significant, though the effect size of weight gain was considerably greater than in the samples for equations 1 and 2. The AUC (95% CI) for the three equations were, equation 1∶86.5% (82.5–90.4%), equation 2∶86.1% (82.8–89.4%) and equation 3∶91.1% (87.8–94.4%).

**Table 3:** The final development models showing factors significantly associated with risk of childhood obesity at 2 years for each equation.

	Equation 1	Equation 2	Equation 3
Birthweight z-score	2.09 (1.59, 2.75)	1.67 (1.36, 2.05)	2.28 (1.64, 3.12)
Weight change z-score	4.45 (3.28, 6.04)	4.48 (3.52, 5.72)	8.80 (5.45, 14.21)
Maternal BMI	1.05 (1.00, 1.09)	1.05 (1.01, 1.09)	
South Asian ethnicity^a^	1.80 (1.05, 3.11)		
Gestational age (<37 weeks)^b^	0.26 (0.07, 0.96)		

Reference categories:

a White British;

b Gestational age ≥37 weeks.

Values are odds ratios with 95% confidence intervals.

#### Validity of the prediction equations

The final multivariable bootstrap model for the equation 1 sample demonstrated statistical significance of birthweight z-score, weight gain z-score and maternal BMI, but not gestational age and ethnicity. However, the AUC (95% CI) for the bootstrapped model was the same as for the development model (85.8% (81.6–90.0%)). The bootstrapped models for equations 2 and 3 retained the same variables as the development models, with no change in AUCs.

Due to insufficient numbers equation 1 could not be validated using ALSPAC data. Equations 2 and 3 had AUCs (95% CI) of 85.0% (81.0–89.1%) and 88.6% (85.2–92.0%) respectively when applied to the ALSPAC sample.

### Prediction Equations used in the App

As birthweight z-score and weight gain z-score were significant predictors of childhood obesity in the development and validation models, they were selected as covariates in the sex-adjusted prediction model for the App. Discrimination accuracy of the risk scores for predicting childhood obesity was excellent, equation 1: AUC 85.3% (95% CI 81.0–89.6%), equation 2: AUC 85.7% (82.4–89.0%) and equation 3: AUC 91.1% (87.9–94.4%). The coefficients used to derive the childhood obesity risk scores from the final multivariable regression model are presented in table 4. At the 10% distribution cut-off point, the ranges from the diagnostic tests for the three equations were: sensitivity (50.6–65.6%), specificity (93.5–94.9%), PPV (41.2–54.1%) and NPV (95.5–96.8%) (Table 5).

**Table 4:** Coefficients used to derive the childhood obesity risk score from a multivariable logistic regression model for each equation comprising baby’s sex, birthweight z-score and weight change z-score.

		Coefficient values		
	Variable	Equation 1	Equation 2	Equation 3
Α	Constant	−3.718	−3.542	−3.937
β1	Female sex	0.488	0.288	0.234
β2	Birthweight z-score	0.599	0.551	0.824
β3	Weight z-score change^a^	1.501	1.508	2.174

a Weight change z-score from birth to 6, 9 or 12 months in equation 1, 2 and 3 samples respectively.

Probability childhood obesity = 1/(1+ e ^-[α+β1+ β2+ β3]^).

**Table 5:** The predictive ability of the obesity risk score for childhood obesity derived from a model comprising baby’s sex, birthweight z-score and weight change z-score between birth and 6 (equation 1), 9 (equation 2) or 12 (equation 3) months.

Proportion of the populationabove risk score threshold (%)	Risk scorethreshold	Sensitivity %(95% CI)	Specificity %(95% CI)	PPV %(95% CI)	NPV %(95% CI)
Equation 1					
30	0.0731	78.3 (67.9, 86.6)	74.2 (71.3, 77.0)	21.2 (16.7, 26.2)	97.5 (96.1, 98.5)
20	0.1155	69.9 (58.8, 79.5)	84.3 (81.9, 86.6)	28.3 (22.2, 35.0)	96.9 (95.5, 98.0)
10	0.2072	50.6 (39.4, 61.8)	93.6 (91.9, 95.1)	41.2 (31.5, 51.4)	95.5 (94.0, 96.8)
Equation 2					
30	0.0662	77.7 (69.2, 84.8)	73.9 (71.5, 76.2)	20.4 (16.8, 24.4)	97.5 (96.3, 98.3)
20	0.1104	68.6 (59.5, 76.7)	84.1 (82.1, 86.0)	27.0 (22.1, 32.4)	96.9 (95.8, 97.8)
10	0.2082	53.7 (44.4, 62.8)	93.5 (92.1, 94.8)	41.7 (33.8, 49.8)	95.9 (94.7, 96.9)
Equation 3					
30	0.0609	86.9 (75.8, 94.2)	75.1 (71.6, 78.3)	24.1 (18.6, 30.3)	98.4 (96.9, 99.3)
20	0.1065	77.0 (64.5, 86.8)	85.1 (82.1, 87.7)	32.0 (24.5, 40.2)	97.6 (96.0, 98.7)
10	0.2391	65.6 (52.3, 77.3)	94.9 (93.0, 96.5)	54.1 (42.1, 65.7)	96.8 (95.2, 98.0)

PPV = positive predictive value; NPV = negative predictive value.

The coefficients used to derive the risk scores for a further three models which additionally included maternal BMI are presented in table 6. The ranges from the diagnostic tests at the 10% cut-off point were: sensitivity (50.6–67.2%), specificity (93.5–95.1%), PPV (40.8–55.4%) and NPV (95.5–97.0%) (Table 7). The AUC (95% CI) for equations 1, 2 and 3 were 85.8% (81.6–90.0%), 86.1% (82.8–89.4%) and 91.1% (87.9–94.4%) respectively.

**Table 6:** Coefficients used to derive the childhood obesity risk score from a multivariable logistic regression model for each equation comprising baby’s sex, birthweight z-score, weight change z-score and maternal BMI. Probability childhood obesity = 1/(1+ e ^-[α+β1+ β2+ β3]+β4^).

		Coefficient values		
	Variable	Equation 1	Equation 2	Equation 3
α	Constant	−4.920	−4.745	−4.625
β1	Female sex	0.493	0.255	0.230
β2	Birthweight z-score	0.577	0.505	0.798
β3	Weight change z-score^a^	1.494	1.501	2.149
β4	Maternal BMI	0.044	0.046	0.026

a Weight change z-score from birth to 6, 9 or 12 months in equation 1, 2 and 3 samples respectively.

**Table 7:** The predictive ability of the obesity risk score for childhood obesity derived from a model comprising sex, birthweight z-score, weight change z-score between birth and 6 (equation 1), 9 (equation 2) or 12 (equation 3)months, and maternal BMI.

Proportion of the populationabove risk score threshold (%)	Risk scorethreshold	Sensitivity %(95% CI)	Specificity %(95% CI)	PPV %(95% CI)	NPV %(95% CI)
Equation 1					
30	0.0696	81.9 (72.0, 89.5)	74.5 (71.6, 77.3)	22.1 (17.6, 27.2)	97.9 (96.6, 98.8)
20	0.1156	71.1 (60.1, 80.5)	84.5 (82.0, 86.7)	28.8 (22.7, 35.5)	97.1 (95.7, 98.1)
10	0.2183	50.6 (39.4, 61.8)	93.5 (91.7, 95.0)	40.8 (31.2, 50.9)	95.5 (94.0, 96.8)
Equation 2					
30	0.0646	76.9 (68.3, 84.0)	74.0 (71.6, 76.3)	20.3 (16.7, 24.2)	97.4 (96.2, 98.3)
20	0.1076	69.4 (60.4, 77.5)	84.2 (82.2, 86.1)	27.5 (22.5, 32.8)	97.0 (95.9, 97.9)
10	0.2042	52.1 (42.8, 61.2)	93.7 (92.3, 94.9)	41.4 (33.5, 49.7)	95.8 (94.6, 96.8)
Equation 3					
30	0.0612	88.5 (77.8, 95.3)	75.2 (71.8, 78.5)	24.5 (19.0, 30.8)	98.6 (97.2, 99.4)
20	0.1051	77.0 (64.5, 86.8)	85.2 (82.3, 87.8)	32.2 (24.7, 40.4)	97.6 (96.0, 98.7)
10	0.2404	67.2 (54.0, 78.7)	95.1 (93.2, 96.6)	55.4 (43.4, 67.0)	97.0 (95.3, 98.1)

PPV = positive predictive value; NPV = negative predictive value.

### The App: Healthy Infant Weight?

**Figure 1:**
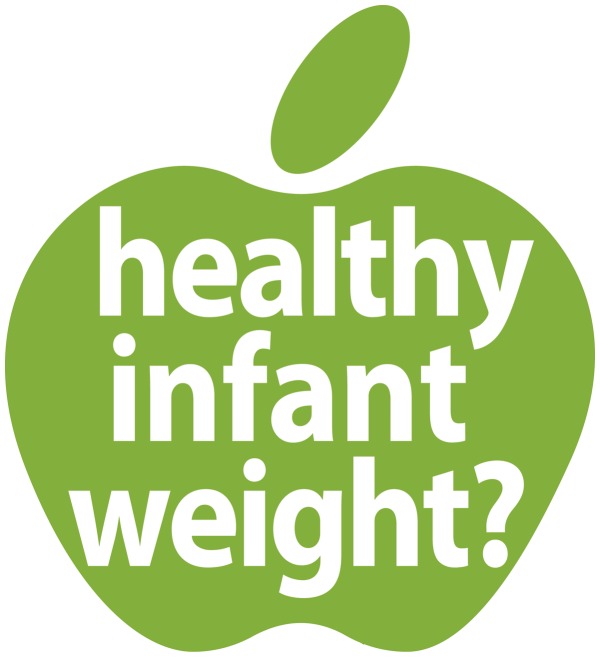
The *Healthy Infant Weight*? App.

Figure 1 shows the *Healthy Infant Weight?* App icon. Baby’s sex, date of birth, birthweight and current weight are required, and maternal height and weight (to calculate BMI) are optional. The App can accept weight measurements in kilograms or pounds and height in centimetres or feet and inches. A risk assessment is displayed as high, medium or low risk of obesity and the current weight z-score is displayed. We chose a risk score distribution cut-off threshold of 10% as being high risk as this approximately reflected the proportion of children in our development and validation samples with obesity and rapid weight gain at 2 years. Children with a cut-off threshold of between 10–20% were defined as being of medium risk and children above 20% low risk. The risk assessment page is accompanied by government endorsed advice on healthy eating, physical activity and parenting tips together with links to an external website where further information can be obtained.

The App can be used on all Apple devices (iPhone, iPad and iPod Touch) and is free to download from the App store.

## Discussion

Childhood obesity is a major public health threat in the UK [1] and innovative strategies to identify infants at the greatest risk are necessary for its prevention. Here, we provide proof of concept that childhood obesity risk prediction equations can be developed using existing birth cohort data and incorporated into a mobile phone application, suitable for use by parents and health care practitioners. The resulting App allows the prediction of risk for childhood obesity during a critical 9 month period of early growth (4.5 to 13.5 months), when biological responses to environmental stimuli can initiate obesogenic trajectories that have long-term consequences for health [30].

There is extensive literature on the early life risk factors for obesity [10]–[12], which is summarised in a recent review of systematic reviews [31]. Along with maternal diabetes, maternal smoking, no or short duration of breastfeeding, short sleep duration and physical inactivity; high birthweight and rapid infant weight gain were identified as consistent predictors of high obesity risk. The systematic review of Baird et al [32], for example, showed an increased odds of obesity at ages 4.5–20 years in infants who grew the fastest ranged between 1.06 and 5.70. These odds ratios were generally greater than those for the other risk factors [31], but this is perhaps not surprising given that infant growth is a surrogate measure of accumulation of risk because the other risk factors act to accelerate early life growth to put infants on a trajectory towards obesity [33]–[37]. This is why our prediction equations focused on weight gain as the key predictor of risk for childhood obesity.

A recent meta-analysis of 10 birth cohort studies including 47,661 participants reported that a one unit increase in weight z-score change between birth and one year of age conferred a two-fold increase in risk for childhood obesity at ages between six and 14 years [15]. This study had a large multi-national sample and an outcome in middle to late childhood, but it only provides an equation to predict the risk of childhood obesity at one year of age. In this way, it is similar to other smaller studies that have used logistic regression to investigate exposures that confer increased childhood obesity risk [13], [17], [38]–[40]. The problem with these existing prediction equations is that there is no practical translation as we cannot expect all infants to be assessed for obesity risk at one year of age, for example, or to have the same variables collected at the assessment as included in the prediction equations.

A paper-based tool to predict an infant’s risk of childhood obesity has been proposed [17], but it relies heavily on the user and has a number of design issues. For example, the equation requires conditional weight gain, thus the user has to convert raw data to z-scores, which necessitates a strong understanding of statistics. As an obesity prediction tool needs to incorporate multiple complex equations and perform background calculations whilst also being user friendly, we believe that an electronic tool is the only realistic option. Indeed, a web-based prediction tool has recently been developed [18] which performs the background calculations and is therefore a great improvement on the paper-based model. The tool requires information on parental BMI, number of household members, maternal professional category, gestational smoking and birth weight, and this requirement for so many variables may unfortunately limit the usability of the tool. During the development of our App, we found that maternal BMI was often not available, and focus groups with health visitors revealed that frequently this was because the mother was unwilling to reveal her weight or be weighed. This is why we chose to have maternal BMI as an optional addition to the App, with little or no difference in discrimination. Furthermore, many mothers are not able to provide paternal BMI, either because they are single parents or simply because they did not know it: for example, it is notable that 12% of the BiB cohort sampled in this study were single or not cohabiting with their partner. In addition, it has been reported that women tend to underestimate the weight of partners who are very overweight [41], thus there are several risks of introducing bias when parental BMI is required for prediction either through missingness or error of this information.

We present the development of a practical mobile phone application that can be used during a wide range of ages (4.5 to 13.5 months) in infancy when growth monitoring is part of routine health care [16]. The App requires information on baby’s sex, date of birth, birthweight and current weight, and users can optionally add maternal height and weight (to calculate BMI). We chose not to include ethnicity and gestational age in equation 1 because although they were significant predictors in the development model, neither of these factors was significant in the internal and external validity analyses. Furthermore, ethnicity in our sample was restricted to White British and South Asian and it was felt that this would not reflect the ethnic diversity (or lack thereof) in many areas. The App is user friendly; it requests only essential information, allows the user to input data in any unit, and is designed so the user is always moving forward without having to return to screens that they have already seen. The advantages of an electronic tool over alternatives, such as the paper based tool [17], are that the App incorporates complex background equations to avoid the practitioner having to do the calculations themselves; it can include any number of prediction equations to account for infants being assessed at different ages and for the real life scenario that not all predictor variables will be available in all instances, and it gives a simple result linked to evidence based advice.

The lack of a hard outcome in adolescence or adulthood is perhaps the greatest limitation of the present study, because even though obesity risk tracks across the life course [42]–[46], some infants with high risk for our outcome at age two years may not progress to develop childhood obesity or disease outcomes in adolescence or adulthood. We did, however, include a measure of rapid weight gain over the prior age period (>1 centile band between 0–2 years) in our outcome because it is a major risk factor for a plethora of adverse health outcomes [13]–[15], [47]–[50] and therefore has greater specificity than just high BMI. Another limitation is that although the prediction equations were developed in the UK in a predominantly White/South Asian cohort in an area with high levels of socio-economic deprivation, and validated in a separate predominantly White cohort with low levels of socio-economic deprivation also in the UK, the equations may not be generalisable to international populations; further validation is therefore warranted.

As a next step, qualitative work is needed to understand how this tool will be received by health care practitioners. As children in the Born in Bradford cohort grow up there will be an opportunity to update our prediction equations using later life health outcomes. Alternatively, a similar approach to that used in the Druet et al [15] paper could be used to pool data from UK and international birth cohort studies to develop a series of prediction equations for use in infancy. Now the App has been developed, new prediction equations can be incorporated as software updates with minimal work. The App could also be developed to incorporate any number of other functionalities, such as plotting of growth measurements on a growth chart, geospatial mapping of an infant’s obesity risk score compared to their peers, and an obesity prevention programme for those infants identified as having high risk. A social networking service could also be integrated into the App to encourage parents to share personal experiences and learn from one another. Social networking analysis suggests that an individual’s weight is influenced by their friendship network [51] and this may be a factor in the spread of obesity [52], particularly in environments where overweight and obesity is prevalent and there is a misperception of one’s own weight status [53]. Thus, the use of the App may become more widespread as parents discuss it within their social networks, resulting in a greater awareness of the risk of childhood obesity and possibly the sharing of information on ways to prevent it. Other future developments include building a web-based application, which could be integrated into existing clinical software, and to create an App for the android platform, thereby allowing the App to be accessed by a wider audience.

In conclusion, we have developed data driven prediction equations for risk of childhood obesity and incorporated them into a mobile phone application, thereby providing proof of concept that childhood obesity prediction research can be integrated with advancements in technology to deliver a clinically relevant tool to practitioners.

## References

[1] Wang Y, Lobstein T (2006) Worldwide trends in childhood overweight and obesity. IntJPediatrObes 1: 11–25. 10.1080/17477160600586747 17902211

[2] McPherson K, Marsh T, Brown M (2007) Modelling Future Trends in Obesity and the Impact on Health.

[3] Bundred P, Kitchiner D, Buchan I (2001) Prevalence of overweight and obese children between 1989 and 1998: population based series of cross sectional studies. BMJ 322: 326–328. 11159654 10.1136/bmj.322.7282.326 PMC26573

[4] Kimbro RT, Brooks-Gunn J, McLanahan S (2007) Racial and ethnic differentials in overweight and obesity among 3-year-old children. AmJPublic Health 97: 298–305. 10.2105/AJPH.2005.080812 PMC1781385 17194857

[5] Lobstein T, Baur L, Uauy R (2004) Obesity in children and young people: a crisis in public health. ObesRev 5 Suppl 14–104. 10.1111/j.1467-789X.2004.00133.x 15096099

[6] Freedman DS, Khan LK, Dietz WH, Srinivasan SR, Berenson GS (2001) Relationship of childhood obesity to coronary heart disease risk factors in adulthood: the Bogalusa Heart Study. Pediatrics 108: 712–718. 11533341 10.1542/peds.108.3.712

[7] Singh AS, Mulder C, Twisk JW, van Mechelen W, Chinapaw MJ (2008) Tracking of childhood overweight into adulthood: a systematic review of the literature. Obes Rev 9: 474–488. 18331423 10.1111/j.1467-789X.2008.00475.x

[8] Han JC, Lawlor DA, Kimm SY (2010) Childhood obesity. Lancet 375: 1737–1748. 20451244 10.1016/S0140-6736(10)60171-7 PMC3073855

[9] Procter KL (2007) The aetiology of childhood obesity: a review. NutrResRev 20: 29–45. 10.1017/S0954422407746991 19079859

[10] Lawlor DA, Chaturvedi N (2006) Treatment and prevention of obesity–are there critical periods for intervention? IntJEpidemiol 35: 3–9. 10.1093/ije/dyi309 16396899

[11] Lawlor DA, Lichtenstein P, Langstrom N (2011) Association of maternal diabetes mellitus in pregnancy with offspring adiposity into early adulthood: sibling study in a prospective cohort of 280,866 men from 248,293 families. Circulation 123: 258–265. 21220735 10.1161/CIRCULATIONAHA.110.980169 PMC4440894

[12] Parsons TJ, Power C, Manor O (2001) Fetal and early life growth and body mass index from birth to early adulthood in 1958 British cohort: longitudinal study. BMJ 323: 1331–1335. 11739217 10.1136/bmj.323.7325.1331 PMC60670

[13] Reilly JJ, Armstrong J, Dorosty AR, Emmett PM, Ness A, et al (2005) Early life risk factors for obesity in childhood: cohort study. BMJ 330: 1357. 15908441 10.1136/bmj.38470.670903.E0 PMC558282

[14] Singhal A, Fewtrell M, Cole TJ, Lucas A (2003) Low nutrient intake and early growth for later insulin resistance in adolescents born preterm. Lancet 361: 1089–1097. 12672313 10.1016/S0140-6736(03)12895-4

[15] Druet C, Stettler N, Sharp S, Simmons RK, Cooper C, et al (2012) Prediction of childhood obesity by infancy weight gain: an individual-level meta-analysis. PaediatrPerinatEpidemiol 26: 19–26. 10.1111/j.1365-3016.2011.01213.x 22150704

[16] Department of Health (2008) Healthy weight, healthy lives: a cross-government strategy for England. London, UK: Department of Health.

[17] Rudolf M (2011) Predicting babies’ risk of obesity. British Medical Journal 96: 995–997. 10.1136/adc.2010.197251 21828069

[18] Morandi A, Meyre D, Lobbens S, Kleinman K, Kaakinen M (2012) Estimation of newborn risk for child or adolescent obesity: lessons from longitudinal birth cohorts. Plos One 7. 10.1371/journal.pone.0049919 PMC3509134 23209618

[19] Horowitz B (2011) 75% of Physicians Prefer Apple iPad, iPhone: Survey. eWeek.

[20] Wright J, Small N, Raynor P, Tuffnell D, Bhopal R, et al.. (2012) Cohort Profile: The Born in Bradford multiethnic family cohort study. International Journal of Epidemiology In press. 10.1093/ije/dys112 23064411

[21] Raynor P (2008) Born in Bradford, a cohort study of babies born in Bradford, and their parents: protocol for the recruitment phase. BMCPublic Health 8: 327. 10.1186/1471-2458-8-327 PMC2562385 18811926

[22] Bryant M, Santorelli G, Fairley L, West J, Lawlor DA, et al.. (2013) Design and characteristics of a new birth cohort, beginning in pregnancy, to study the early origins and ethnic variation of childhood obesity: the BiB1000 study. Longitudinal and Life Course Studies. 4(2): 119–135, 2013.

[23] Johnson W, Cameron N, Dickson P, Emsley S, Raynor P, et al (2009) The reliability of routine anthropometric data collected by health workers: a cross-sectional study. IntJNursStud 46: 310–316. 10.1016/j.ijnurstu.2008.10.003 19019368

[24] Howe LD, Tilling K, Lawlor DA (2009) Accuracy of height and weight data from child health records. ArchDisChild 94: 950–954. 10.1136/adc.2009.162552 19689966

[25] Freeman JV, Cole TJ, Chinn S, Jones PR, White EM, et al (1995) Cross sectional stature and weight reference curves for the UK, 1990. ArchDisChild 73: 17–24. 10.1136/adc.73.1.17 PMC1511167 7639543

[26] Cameron N, Preece MA, Cole TJ (2005) Catch-up growth or regression to the mean? Recovery from stunting revisited. AmJHumBiol 17: 412–417. 10.1002/ajhb.20408 15981181

[27] StataCorp (2009) *Stata Statistical Software: Release 11*. College Station, TX: StataCorp LP.

[28] Efron B, Tibshirani R (1993) An introduction to the bootstrap. Monographs on statistics and applied probablility. New York: Chapman & Hall.

[29] Boyd A, Golding J, Macleod J, Lawlor DA, Fraser A, et al.. (2012) Cohort Profile: The ‘Children of the 90s’–the index offspring of the Avon Longitudinal Study of Parents and Children. Int J Epidemiol. 10.1093/ije/dys064 PMC3600618 22507743

[30] Cameron N, Demerath EW (2002) Critical periods in human growth and their relationship to diseases of aging. AmJPhysAnthropol Suppl 35: 159–184. 10.1002/ajpa.10183 12653312

[31] Monasta L, Batty GD, Cattaneo A, Lutje V, Ronfani L, et al (2010) Early-life determinants of overweight and obesity: a review of systematic reviews. ObesRev 11: 695–708. 10.1111/j.1467-789X.2010.00735.x 20331509

[32] Baird J, Fisher D, Lucas P, Kleijnen J, Roberts H, et al (2005) Being big or growing fast: systematic review of size and growth in infancy and later obesity. BMJ 331: 929. 16227306 10.1136/bmj.38586.411273.E0 PMC1261184

[33] Baptiste-Roberts K, Nicholson WK, Wang NY, Brancati FL (2012) Gestational diabetes and subsequent growth patterns of offspring: the National Collaborative Perinatal Project. Matern Child Health J 16: 125–132. 21327952 10.1007/s10995-011-0756-2 PMC3707279

[34] Betoko A, Charles MA, Hankard R, Forhan A, Bonet M, et al.. (2012) Determinants of infant formula use and relation with growth in the first 4 months. MaternChild Nutr. 10.1111/j.1740-8709.2012.00415.x PMC6860242 22642271

[35] Crume TL, Ogden L, Daniels S, Hamman RF, Norris JM, et al (2011) The impact of in utero exposure to diabetes on childhood body mass index growth trajectories: the EPOCH study. JPediatr 158: 941–946. 21238981 10.1016/j.jpeds.2010.12.007 PMC3090715

[36] Lampl M, Johnson ML (2011) Infant growth in length follows prolonged sleep and increased naps. Sleep 34: 641–650. 21532958 10.1093/sleep/34.5.641 PMC3079944

[37] Suzuki K, Kondo N, Sato M, Tanaka T, Ando D, et al (2012) Maternal smoking during pregnancy and childhood growth trajectory: a random effects regression analysis. JEpidemiol 22: 175–178. 22277789 10.2188/jea.JE20110033 PMC3798597

[38] Koshy G, Delpisheh A, Brabin BJ (2011) Dose response association of pregnancy cigarette smoke exposure, childhood stature, overweight and obesity. EurJPublic Health 21: 286–291. 10.1093/eurpub/ckq173 21126981

[39] Oken E, Levitan EB, Gillman MW (2008) Maternal smoking during pregnancy and child overweight: systematic review and meta-analysis. Int J Obes (Lond) 32: 201–210. 18278059 10.1038/sj.ijo.0803760 PMC2586944

[40] Taveras EM, Gillman MW, Kleinman K, Rich-Edwards JW, Rifas-Shiman SL (2010) Racial/ethnic differences in early-life risk factors for childhood obesity. Pediatrics 125: 686–695. 20194284 10.1542/peds.2009-2100 PMC3836212

[41] Christensen VT (2012) Gendered perceptions of own and partner weight-level. Health (London) 16: 382–399. 22067916 10.1177/1363459311425512

[42] Freitas D, Beunen G, Maia J, Claessens A, Thomis M, et al (2012) Tracking of fatness during childhood, adolescence and young adulthood: a 7-year follow-up study in Madeira Island, Portugal. AnnHumBiol 39: 59–67. 10.3109/03014460.2011.638322 22148930

[43] Chrzanowska M, Suder A, Kruszelnicki P (2012) Tracking and risk of abdominal obesity in the adolescence period in children aged 7–15. The Cracow Longitudinal Growth Study. AmJHumBiol 24: 62–67. 10.1002/ajhb.22204 22131191

[44] Oellingrath IM, Svendsen MV, Brantsaeter AL (2011) Tracking of eating patterns and overweight - a follow-up study of Norwegian schoolchildren from middle childhood to early adolescence. NutrJ 10: 106. 21978299 10.1186/1475-2891-10-106 PMC3200168

[45] Ge S, Kubota M, Nagai A, Mamemoto K, Kojima C (2011) Retrospective individual tracking of body mass index in obese and thin adolescents back to childhood. Asia PacJClinNutr 20: 432–437. 21859663

[46] Toselli S, Ventrella AR, Brasili P (2012) Prevalence and tracking of weight disorders in Italian primary school students: a three-year follow-up. CollAntropol 36: 63–67. 22816199

[47] Adair LS (2007) Size at birth and growth trajectories to young adulthood. AmJHumBiol 19: 327–337. 10.1002/ajhb.20587 17421008

[48] Kuzawa CW, Hallal PC, Adair L, Bhargava SK, Fall CH, et al (2012) Birth weight, postnatal weight gain, and adult body composition in five low and middle income countries. AmJHumBiol 24: 5–13. 10.1002/ajhb.21227 PMC3541478 22121058

[49] Norris SA, Osmond C, Gigante D, Kuzawa CW, Ramakrishnan L, et al (2012) Size at birth, weight gain in infancy and childhood, and adult diabetes risk in five low- or middle-income country birth cohorts. Diabetes Care 35: 72–79. 22100968 10.2337/dc11-0456 PMC3241316

[50] van der Gugten AC, Koopman M, Evelein AM, Verheij TJ, Uiterwaal CS, et al (2012) Rapid early weight gain is associated with wheeze and reduced lung function in childhood. EurRespirJ 39: 403–410. 10.1183/09031936.00188310 21852338

[51] Ali MM, Amialchuk A, Gao S, Heiland F (2007) Weight Gain and Social Networks: Is There a Contagion Effect? N Engl J Med 257: 370–379.

[52] Christakis NA, Fowler JH (2007) The spread of obesity in a large social network over 32 years. N Engl J Med 357: 370–379. 17652652 10.1056/NEJMsa066082

[53] Ali MM, Amialchuk A, Renna F (2011) Social Network and Weight Misperception among Adolescents. Southern Economic Journal 77: 827–842.

